# Emergency Presentations for Dizziness—Radiological Findings, Final Diagnoses, and Mortality

**DOI:** 10.1155/2023/7450009

**Published:** 2023-06-20

**Authors:** Jeannette-Marie Busch, Isabelle Arnold, Julia Karakoumis, David J. Winkel, Martin Segeroth, Christian H. Nickel, Roland Bingisser

**Affiliations:** ^1^Emergency Department, University Hospital Basel, Basel, Switzerland; ^2^Department of Radiology, University Hospital Basel, Basel, Switzerland

## Abstract

**Background:**

Dizziness is a frequent presentation in patients presenting to emergency departments (EDs), often triggering extensive work-up, including neuroimaging. Therefore, gathering knowledge on final diagnoses and outcomes is important. We aimed to describe the incidence of dizziness as primary or secondary complaint, to list final diagnoses, and to determine the use and yield of neuroimaging and outcomes in these patients.

**Methods:**

Secondary analysis of two observational cohort studies, including all patients presenting to the ED of the University Hospital of Basel from 30th January 2017–19th February 2017 and from 18th March 2019–20th May 2019. Baseline demographics, Emergency Severity Index (ESI), hospitalization, admission to Intensive Care Units (ICUs), and mortality were extracted from the electronic health record database. At presentation, patients underwent a structured interview about their symptoms, defining their primary and secondary complaints. Neuroimaging results were obtained from the picture archiving and communication system (PACS). Patients were categorized into three non-overlapping groups: dizziness as primary complaint, dizziness as secondary complaint, and absence of dizziness.

**Results:**

Of 10076 presentations, 232 (2.3%) indicated dizziness as their primary and 984 (9.8%) as their secondary complaint. In dizziness as primary complaint, the three (out of 73 main conditions defined) main diagnoses were nonspecific dizziness (47, 20.3%), dysfunction of the peripheral vestibular system (37, 15.9%), as well as somatization, depression, and anxiety (20, 8.6%). 104 of 232 patients (44.8%) underwent neuroimaging, with relevant findings in 5 (4.8%). In dizziness as primary complaint 30-day mortality was 0%.

**Conclusion:**

Work-up for dizziness in emergency presentations has to consider a broad differential diagnosis, but due to the low yield, it should include neuroimaging only in few and selected cases, particularly with additional neurological abnormalities. Presentation with primary dizziness carries a generally favorable prognosis lacking short-term mortality. .

## 1. Introduction

Dizziness is among the most common primary complaints at presentation to emergency departments (EDs) [1, 2], and it is often considered a nonspecific complaint [3] due to its extremely broad differential diagnosis [4–8]. As a secondary complaint, dizziness is most often reported in combination with headache, weakness, nausea, and fatigue [9]. Its incidence appears to rise concomitantly with the total number of symptoms at presentation [9].

The work-up of dizziness is complex. First, the differential diagnosis is broad and certainly not limited to the typical central (e.g., ischemia) and peripheral (e.g., BPLS) causes [10], but extends to a multitude of other causes [11]. Second, pretest probabilities regarding individual serious conditions (e.g., cerebrovascular, infectious, and tumorous), morbidity, and mortality are largely unknown in unselected emergency presentations. Third, challenging the common belief, that careful taking of medical history allows to differentiate between dizziness, vertigo, and presyncopal complaints, the use of such descriptors is unreliable [12]—being vague, inconsistent, or contradictory in many patients [13].

Furthermore, the attempt to differentiate between vertigo and dizziness does not seem to support the process of diagnosis [10] and patient recorded vertigo is no more predictive than dizziness for specific diagnoses [14]. In spite of the evidence that patients with isolated dizziness and no other neurologic complaints/findings are safe to be discharged due to low risk for acute cerebrovascular complications [15], extensive evaluations [16], such as computed tomography (CT) and magnetic resonance imaging (MRI) are increasingly used [17], leading to substantial secondary cost [18]. Advanced imaging might be driven by insecurity and fear of malpractice suits [19]. Some authors have described a 50% rate of imaging in patients presenting with dizziness [20], with a very low diagnostic yield [21, 22]. To reduce overuse of imaging, it is of importance to increase knowledge on pretest probabilities (final diagnoses) and outcomes in this notoriously difficult population. Prognostication is important to emergency physicians, as resources are limited and those in need (patients with high risk of serious conditions and critical prognosis) should not have to compete for imaging resources, hospital beds, or caregiver time.

We have therefore planned this secondary analysis combining the data of two prospective studies. These cohorts included all patients consecutively presenting to an academic ED in order to describe the incidence of dizziness as primary or secondary complaint, the use of resources (e.g., specialist consultations and neuroimaging), the use and yield of neuroimaging, and outcomes, such as final diagnoses, hospitalization, intensive care, and mortality.

## 2. Methods

### 2.1. Study Design and Setting

Analysis of a monocentric prospective all-comer observational study, consisting of two separate cohorts, conducted as a quality control at the Emergency Department of the University Hospital of Basel.

The ED of the University Hospital of Basel is a tertiary academic center and has a census of over 50,000 patients a year. Obstetric, pediatric, and ophthalmologic patients are treated elsewhere. All patients presenting were included 24/7 between February 1st and February 23rd, 2015 and between March 18th 2019 and May 20th, 2019.

### 2.2. Data Collection

All patients presenting to the ED of the University Hospital of Basel during the time periods described were included by a medically trained study team. After triage, using the Emergency Severity Index (ESI, Supplementary Table 1) [23], patients were interviewed regarding all of their symptoms at presentation. In a second step, they were asked about their primary complaint leading to ED presentation. Dizziness and vertigo were both recorded as dizziness, as there is no distinction in German. The study team also recorded routine vital sign measurements, such as heart rate, blood pressure, level of consciousness (as determined by the AVPU Scale), body temperature, peripheral oxygen saturation, and respiratory rate. Baseline characteristics such as age, sex, diagnosis at ED discharge, consultations by specialists, hospital diagnoses, and hospitalization, as well as admission to the Intensive Care Unit (ICU) were obtained from the electronic health record (EHR) provided by Protect Data®, Boswil, Switzerland, and the International Statistical Classification of Diseases and related health problems (ICD) coding tool.

In order to obtain an expert attributed ICD code of the final ED diagnosis, we performed a structured chart review, complying with 7 of 8 points according to Gilbert et al. [24]. Two physicians, with ten and one year of clinical experience, respectively, independently attributed ICD codes after chart abstraction. In case of disagreement, the results of the independent chart reviews were discussed among the authors, using a modified Delphi method. The initial interrater reliability (IRR) between reviewers was 64.9% regarding the three digit ICD-10 code.

In cases with dizziness as primary complaint, all information obtained by neuroimaging was reviewed and rated for “relevant findings.” Relevant findings were defined as “major abnormalities or critical findings that affected management or demanded direct reporting to the responsible physician” [25].

All advanced imaging data were obtained from the picture archiving and communication system (PACS). Using PACS crawlers, all neuroimaging, computed tomography (CT), and magnetic resonance imaging (MRI), obtained within 24 hours of presentation, were registered.

Diagnostic groups for final diagnoses were based on ICD-10 codes at discharge (Supplementary Table 2).

### 2.3. Follow-Up

Follow-up regarding survival was conducted by query of the official registries in Switzerland. In cases lacking Swiss social security numbers, patients were checked for representation within a year, or contacted directly. If the respective patient could not be contacted, proxies or primary care providers were interviewed by telephone or questionnaires.

### 2.4. Patient Selection

All patients presenting to the ED during the study period were eligible for inclusion. Patients were divided into three nonoverlapping groups: (a) patients presenting with dizziness as primary complaint, (b) patients presenting with dizziness as secondary complaint, and (c) nondizzy patients.

### 2.5. Study Aims

The aim of this study was to determine the incidence of dizziness (primary and secondary) in an all-comer ED population and to describe demographics; use and yield of neuroimaging; number of consultations by neurology and neurosurgery (aggregated to neurology) or ear, nose, and throat (ENT) specialists; final diagnoses; and outcomes (hospitalization, intensive care, and mortality).

### 2.6. Definitions

Relevant findings by advanced imaging were categorized to tumor, ischemia, subdural hematoma, infarction, and intracerebral hemorrhage.

Due to re-presentations, different populations were used for descriptive and inferential statistics. The term “patient” was used and defined as the first presentation of each patient, while the term “presentation” was used and defined as all presentations (first and repeated presentation) of each patient.

Admission was defined as hospitalization to any ward, including geriatrics, palliative care, or intensive care (ICU).

Primary complaint was defined as the main reason the patient presented to the ED. Secondary complaint was defined as any complaint patients mentioned in the interview at presentation, excluding the main reason for presentation.

### 2.7. Statistical Methods

For statistical analysis, the software R (Version 4.1.2 [26]) was used. Continuous variables were compared using the Student *t*-test; categorical variables were compared using the chi [2] test.

Outcomes were assessed by including the first presentation of each patient, and odds ratios (OR) were calculated using logistic regressions with nondizzy patients as reference, corrected for age and sex.

Statistical significance was defined as *p* < 0.05; confidence intervals (CI) were set at 95%.

### 2.8. Ethics

This study was approved by the local Ethics Committee (identifier 236/13, ww.eknz.ch, amendment for prolongation PB_2019_00008), and conducted according to the principles of the Declaration of Helsinki. Written informed consent was waived due to the observational nature of the study. Oral informed consent was noted in all patients' EHR. Patients were excluded if the EHR was marked with “rejection to participate in research,” or if patients orally declined participation.

## 3. Results

**Figure 1:**
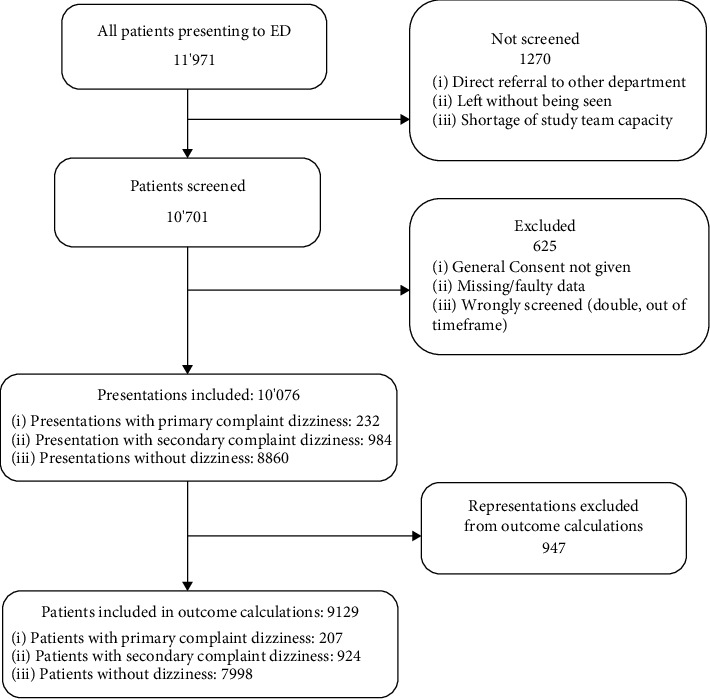
Recruitment and inclusion procedure. ^*∗*^ED: emergency department.

Of 10076 presentations to the ED, 232 (2.3%) presented with dizziness as primary complaint, 984 (9.8%) with dizziness as secondary complaint, and 8860 did not report dizziness (Figure 1).

The three groups were similarly distributed regarding age and sex, but differed in the mean number of symptoms, ESI distribution, and the use of neuroimaging. The mean number of symptoms in dizziness as primary complaint was 2.6 (±1.4), 4.3 (±2.5) in secondary dizziness, and 1.9 (±1.5) in nondizzy presentations. Of 232 primary dizziness presentations, 104 (44.8%) underwent neuroimaging, of those 49 recieved a MRI and 20 (8.6%) both CT and MRI. Of the 984 secondary dizziness presentations, 243 (24.7%) underwent neuroimaging, 36 (3.7%) underwent both CT and MRI, and of the 8860 nondizzy presentations, 1200 (13.5%) underwent neuroimaging, 124 (1.4%) underwent both CT and MRI (Table 1). A relevant finding was identified in five (4.8%) of 104 images, four (8.2%) of the 49 MRIs, and one (5%) of the 20 cases undergoing both imaging modalities (Table 2). The 35 CT scans did not identify a single relevant finding. Signs of leukoencephalopathy was found in 24 (23.1%) of 104, and signs of previous ischemia (“old infarctions”) in 11 (10.6%) of 104 (Table 2). Of the 232 presentations with dizziness as chief complaint, 28 (12.1%) underwent an ENT consultation, 53 (22.4%) a neurology consultation, and 31 (13.4%) patients underwent both. Of the 84 presentations with a neurology consultation, 36 (42.9%) had an MRI, 15 (17.9%) a CT, 15 (17.9%) had both, and 18 (21.4%) did not undergo imaging. Of 120 presentations without consultation, 23 (19.2%) had a CT, five (4.2%) had an MRI, and one (0.8%) underwent both. Relevant findings were identified in three (7.6%) of the 39 presentations undergoing neurology consultation combined with advanced imaging and in two (6.9%) of the 29 presentations receiving advanced imaging with no specialist consultations. Of the 31 patients undergoing both specialist consultations, three (9.6%) had a CT, 18 (58.1%) had an MRI, six (19.6%) underwent both, and in four (12.9%) no advanced imaging was performed. No relevant findings were recorded in the 31 presentations with double consultation (Table 3).The top three final diagnoses of the 232 presentations with dizziness as primary complaint were nonspecific dizziness in 47 (20.3%) presentations, dysfunction of the peripheral vestibular system in 37 (15.9%), and somatization, depression, and anxiety in 20 (8.6%) presentations. Presentations with nonspecific dizziness (final diagnosis) underwent neuroimaging in 26/47 cases (55.3%). The three main final diagnoses were responsible for 44.8% of all diagnoses in primary dizziness, and the 10 most frequent final diagnoses were responsible for 62.4% of all 1216 diagnoses for primary or secondary dizziness. For presentations with dizziness as secondary complaint, the three top final diagnoses were trauma related in 138 (14.0%), “other” in 118 (12.0%), and throat and chest pain in 62 (6.3%); nonspecific dizziness was found in 58 (9.8%) of all presentations with secondary dizziness. Cerebrovascular ischemia was identified in 25 (2.5%) of all presentations with secondary dizziness. Of the nondizzy presentations, the main three final diagnoses were trauma related in 2135 (24.1%), “other” in 1750 (19.8%), and throat and chest pain in 424 (4.8%). Acute cerebral ischemic disease was the final diagnosis in 78 (0.9%) of these presentations (Table 4).Outcomes for hospitalization and 30- and 100-day mortality differed significantly between the three groups (for mortality calculations, repeat presentations were excluded, causing different group sizes): 30-day mortality was 0/207 (0.0%) in primary dizziness, 12/924 (1.3%) in secondary dizziness, and 197/7998 (2.6%) in nondizzy patients (*p* = 0.006). 100-day mortality was 2/207 (1.0%) in primary dizziness, 20/924 (2.2%) in secondary dizziness, and 315/7998 (4.1%) in nondizzy patients (*p* = 0.002) (Table 5).

For 100-day mortality, the OR for dizziness as a primary complaint was 0.18 (CI 0.03/0.77 and *p* = 0.02), and 0.4 (CI 0.18/0.79 and *p* = 0.02) for 1-year mortality (Table 6).

## 4. Discussion

The major findings of the study were the relatively high prevalence of dizziness as primary or secondary complaint, the high use of resources (consultations and imaging), the low prevalence of relevant findings, the predominance of nonspecific dizziness as the final diagnosis, and the favorable outcomes (short- and long-term mortality).

While dizziness as the main presenting complaint has been shown to be among the more frequent in several studies [27, 28], the relatively high incidence of dizziness as a secondary or accompanying complaint is a rather new finding [9]. In spite of inherent uncertainties, both patients and caregivers may have regarding “primary” or “accompanying” complaint [29], this classification is decisive for the subsequent work-up. Secondary dizziness seems to be neglected frequently, due to the “physician filter” [5, 30], or deemed less meaningful (carrying less diagnostic information) due to the importance and predominance of the primary complaint (such as trauma or chest pain).

Therefore, in this prospective all-comer study, we focused on work-up and outcomes in dizziness as a primary complaint. Almost half of all patients underwent consultations and neuroimaging. While patients referred to ENT after ED work-up underwent imaging only in a minority (18%), patients referred to neurology underwent imaging in the majority (76%), and nonreferred patients in 24% of all cases. Relevant imaging findings (e.g., acute ischemia or intracranial hemorrhage) were found in patients with neurology consultations or no consultations—overall in less than 5% of all cases. Of note, in patients presenting with dizziness as accompanying/secondary complaint, the clinical diagnostic group of cerebral ischemia or hemorrhage was of similar relative size as in primary complaints. This finding may challenge the common belief that only patients with primary dizziness should undergo a specific work-up, including neuroimaging. Admittedly, these two groups cannot be directly compared due to lower age, higher acuity, and higher short-term mortality in dizziness as accompanying complaint. Although such findings and comparisons cannot be used for direct explanations of differences, the mere observation may be used to generate new hypotheses. Such hypotheses could be the ground for observational or interventional trials, e.g., comparing diagnostic effectiveness of imaging, or identifying risk factors for acute ischemia in both primary and secondary dizziness.

Previous findings have focused on the identification of acute cerebrovascular ischemia in ED patients presenting with dizziness, with documented prevalence's between 2 and 16% [31–34]. However, there is a high risk of inclusion bias, particularly in studies of retrospective nature, performed by specialists, or subject to a sophisticated inclusion process [33]. It was previously shown that white matter abnormalities in “unexplained dizziness” were more frequent than in patients with an alternative explanation (22% vs. 5%). Particularly in older adults, such findings are common. It remains controversial if they contribute to these complaints, particularly if they are acute, and there is no evidence for specific treatment [35]. Other possible explanations for the very low yield of neuroimaging may be MR-negative transient ischemic attacks (TIA) [36, 37], the use of the wrong modality (particularly CT in a younger population) [38–40], or the high prevalence of serious medical conditions [10] and medications [41], or the low prevalence of acute morbidity [42], depending on the populations and the environment investigated. However, our findings are in line with previous reports showing that discharge of patients with primary dizziness is safe due to a low rate of cerebrovascular ischemia during follow-up [15], and that the use of neuroimaging, particularly CT, is questionable [43] in patients presenting to the ED with primary dizziness.

## 5. Limitations

The study was performed in a single center—excluding patients with eye problems, who might also suffer from dizziness—but were treated elsewhere.

As not every patient received advanced imaging, TIA or cerebral ischemia could have been missed. However, patients with relevant subsequent disabilities would likely have presented to our hospital—being the only stroke center in Northwestern Switzerland.

## 6. Conclusion

Taken together, work-up for dizziness in emergency presentations has to consider a broad differential diagnosis, but due to the low yield, it should include neuroimaging only in few and selected cases, particularly with additional neurological abnormalities. Presentation with primary dizziness carries a generally favorable prognosis lacking short-term mortality. Therefore, ambulatory work-up in patients with ED presentation for dizziness should be considered, and computed tomography should not be used in younger patients.

## Figures and Tables

**Table 1:** Demographics of dizziness at ED presentation.

	Primary complaint (*n* = 232, 2.3%)	Secondary complaint (*n* = 984, 9.8%)	Nondizzy (*n* = 8860, 87.9%)	SMD	*p*
Age (mean (SD))	59.0 (20.3)	54.6 (21.7)	53.1 (21.8)	0.188	<0.001
Sex: Female (*n* (%))	123 (53.0)	557 (56.6)	4094 (46.2)	0.139	<0.001
ESI (*n* (%))				0.496	<0.001
1	1 (0.4)	10 (1.0)	202 (2.3)		
2	67 (28.9)	364 (37.0)	2198 (24.8)		
3	142 (61.2)	464 (47.2)	3444 (38.9)		
4	22 (9.5)	142 (14.4)	2745 (31.0)		
5	0 (0)	3 (0.3)	263 (3.0)		
NA		1 (0.1)	8 (0.0)		
Number of symptoms (mean (SD))	2.6 (1.4)	4.3 (2.5)	1.9 (1.5)	0.824	<0.001
Imaging (*n* (%))				0.544	<0.001
CT	35 (15.1)	165 (16.7)	934 (10.5)		
MRI	49 (21.1)	42 (4.3)	142 (1.6)		
MRI + CT	20 (8.6)	36 (3.7)	124 (1.4)		
None	128 (55.2)	741 (75.3)	7660 (86.5)		
Admission (*n* (%))	94 (40.5)	390 (39.6)	3006 (33.9)	0.091	<0.001

Data are shown as mean and SD for continuous variables and as count and percentage for categorical variables. ^*∗*^SD: standard deviation; ^*∗*^ESI: emergency severity index.

**Table 2:** CT and MRI in patients with dizziness as primary complaint.

	Relevant findings	Leukoencephalopathy	Signs of previous ischemia
All (*n* = 104)	5 (4.8%)	24 (23.1%)	11 (10.6%)
CT (*n* = 35)	0 (0%)	3 (8.6%)	1 (2.9%)
MRI (*n* = 49)	4 (8.2%)	17 (34.7%)	4 (8.2%)
Both (*n* = 20)	1 (5.0%)	4 (20%)	6 (30%)

Findings, including relevant findings, leukoencephalopathy, and old infarctions, are found in advance imaging of the patients with primary complaint dizziness. ^*∗*^CT: computed tomography; ^*∗*^MRI: magnetic resonance imaging. *n* (%).

**Table 3:** ENT and neurology consultations and associated advanced imaging of the 232 patients with primary complaint dizziness.

	ENT consultation	Neurology consultation	Both	No consultation
	*n* = 28 (12.1%)	*n* = 53 (22.8%)	*n* = 31 (13.4%)	*n* = 120 (51.7%)
CT	0 (%)	12 (22.6%)	3 (9.7%)	23 (19.2%)
MRI	4 (14.3%)	18 (34.0%)	18 (58.0%)	5 (4.2%)
MRI + CT	1 (3.6%)	9 (17.0%)	6 (19.4%)	1 (0.8%)
None	23 (82.1%)	14 (26.4%)	4 (12.9%)	91 (75.8%)
Relevant findings	0	3	0	2

This table shows the number of ENT or neurology consultations that the 232 patients with chief complaint dizziness received, as well as the advanced imaging of each group. Also listed are relevant findings in the advanced imaging of each group. The group with no neurology consultation is composed of the group neither and ENT consultations. ^*∗*^CT: computed tomography; ^*∗*^MRI: magnetic resonance imaging; ^*∗*^ENT: ear nose throat. *n* (%).

**Table 4:** Prevalence of final clinical diagnoses.

	Primary complaint	Secondary complaint	Nondizzy
	(*n* = 232, 2.3%)	(*n* = 984, 9.8%)	(*n* = 8860, 87.9%)
Nonspecific dizziness (R42)	47 (20.3%)	58 (5.9%)	15 (0.2%)
Dysfunction of the peripheral vestibular system (H81)	37 (15.9%)	9 (0.9%)	4 (0.0%)
Somatization disorders, depression, and anxiety (F00–F03, F13–F48, and R53, R54)	20 (8.6%)	32 (3.3%)	280 (3.2%)
Trauma related (S00–S02, S06–S14, and T79)	19 (8.2%)	138 (14.0%)	2135 (24.1%)
Syncope and collapse (R55)	14 (6.0%)	47 (4.8%)	106 (1.2%)
Cerebral hemorrhage or ischemic disease	12 (5.2%)	39 (4.0%)	268 (3.0%)
Cerebral hemorrhage (I61)	1 (0.4%)	1 (0.1%)	10 (0.1%)
Acute ischemic disease (I63 and I64)	3 (1.3%)	25 (2.5%)	78 (0.9%)
Cerebrovascular disease (I67)	1 (0.4%)	3 (0.3%)	149 (1.7%)
Sequelae of cerebrovascular disease (I69)	3 (1.3%)	0	0
TIA (G45 w/out G45.4)	3 (1.3%)	10 (1.0%)	31 (3.5%)
TGA (G45.4)	1 (0.4%)	0	0
Orthostasis (I95 and I98)	11 (4.7%)	5 (0.5%)	4 (0.0%)
Drug related problems, intoxications, and poisoning (F10–F13, T36–T47, T50-T51, T65, and T78)	10 (4.3%)	18 (1.8%)	207 (2.3%)
Arrhythmia (R00 and I44–I49)	9 (3.9%)	44 (4.5%)	114 (1.3%)
Hypertension (I10)	9 (3.9%)	22 (2.2%)	71 (0.8%)
Other	7 (3.0%)	118 (12.0%)	1750 (19.8%)
Acute infections of the upper airway (J00–J11)	3 (1.3%)	59 (6.0%)	220 (2.5%)
Abdominal and pelvic pain (R10)	—	33 (3.4%)	539 (6.1%)
Throat and chest pain (R07)	—	62 (6.3%)	424 (4.8%)
Back pain (M54)	—	16 (1.6%)	345 (3.9%)
Breathing disorders (R06)	—	17 (1.7%)	227 (2.6%)
Disease of the skin (L00–L08)	—	4 (0.4%)	155 (1.7%)
Headache (G44 and R51)	—	33 (3.4%)	112 (1.3%)

The top 10 final diagnoses of the three nonoverlapping presentation groups (*n* = 10076), with the numbers supplemented for top 10 conditions of the other groups. Not a complete list of all possible final diagnoses. *n* (%).

**Table 5:** Outcomes, stratified by groups.

	Primary complaint (*n* = 207, 2.3%)	Secondary complaint (*n* = 924, 10.0%)	Nondizzy patients (*n* = 7998, 87.7%)	*p*
Admission (*n* (%))	82 (39.6)	368 (39.8)	2674 (33.4)	<0.001
ICU admission (*n* (%))	11 (5.3)	38 (4.1)	439 (5.5)	0.212
Mortality (*n* (%))				
30 day	0 (0)	12 (1.3)	197 (2.6)	0.006
100 day	2 (1.0)	20 (2.2)	315 (4.1)	0.002
1 year	8 (3.9)	55 (6.2)	553 (7.2)	0.111

Only the first presentation of each patient was included in the outcome calculations (*n* = 9129), to ensure correct calculations of the mortality rates. Data are shown as mean and SD for continuous variables and as count and percentage for categorical variables. ICU: Intensive Care Unit.

**Table 6:** Odds ratios for outcomes, stratified for groups.

	Odds ratio (CI 95%)			
	Primary complaint	*p*	Secondary complaint	*p*
Admission (*n* (%))	1.27 (1.09/1.48)	0.63	1.07 (1.09/1.44)	0.002
ICU admission (*n* (%))	1.07 (0.59/1.78)	0.81	0.73 (0.52/1.01)	0.07
Mortality (*n* (%))				
30 day	^*∗*^		0.43 (0.26/0.85)	0.02
100 day	0.18 (0.03/0.77)	0.02	0.5 (0.3/0.77)	0.003
1 year	0.40 (0.18/0.79)	0.02	0.79 (0.58/1.06)	0.13

Odd ratio for the outcomes hospital admission, ICU admission, 30 days, 100 days, and 1-year mortality. Reference points are the nondizzy patients. Calculations were performed using only the first presentations (*n* = 9129). ^*∗*^Due to no event, this could not be calculated. ICU: Intensive Care Unit.

## Data Availability

Due to patient confidentiality the data cannot be made available.

## Supplementary Materials

Supplementary Materials Supplementary Table 1: Emergency Severity Index. Supplementary Table 2: diagnoses groups according to ICD codes.
