# Supplementary material for: Emergency Presentations for Dizziness—Radiological Findings, Final Diagnoses, and Mortality
Source: Int J Clin Pract. 2023 Jun 20;2023:7450009. doi: 10.1155/2023/7450009 (PMC10299881; doi:10.1155/2023/7450009)
Supplement: Supplementary Materials — Supplementary Table 1: Emergency Severity Index. Supplementary Table 2: diagnoses groups according to ICD codes. [file 7450009.f1.docx]

| **Supplement Table 1: Emergency Severity Index**  The Emergency Severity Index is a triage tool used to assess acuity and severity of patients presenting to the Emergency Department. | |
| --- | --- |
| 1 | Patient requires immediate life-saving intervention |
| 2 | Patient is in a high risk situation, is disoriented, in severe pain, or vitals are in danger zone |
| 3 | If multiple resources are required to stabilize the patient, but vitals are not in the danger zone |
| 4 | If one resource is required to stabilize the patient |
| 5 | If patient does not require any resources to be stabilized |

| Supplement Table 2: Diagnoses Groups according to ICD Codes  ICD codes in diagnoses groups for the classification of final diagnoses. | | |
| --- | --- | --- |
| ICD-10 Chapter | ICD-10 subgroup | No. of subgroups |
| Chapter I  Certain infectious and parasitic diseases  (A00 – B99) | intestinal (A04, A08-A09)  bacteraemia (A40-A41)  nonspecific infections (B99) | 26  19  1 |
| Chapter II  Neoplasms  (C00-D48) | malignant neoplasm (C15-C92)  benign neoplasm (D18-D32) | 504  101 |
| Chapter III  Diseases of the blood and blood-forming organs and certain disorders involving the immune mechanism (D50-D89) | anaemia (D50-D64) | 88 |
| Chapter IV  Endocrine, nutritional and metabolic diseases  (E00-E90) | hormones (E03 - E06, E21 - E27)  diabetes/hypoglycaemia (E11 - E16)  malnutrition (E40 - E46)  electrolyte disorders (E83, E87)  dehydration (E86) | 82  133  9  27  1 |
| Chapter V  Mental and behavioural disorders (F00-F99) | Somatization disorders, including depression and anxiety (F00 - F03, F13 – F48, R53, R54) | 123 |
| Chapter VI  Diseases of the nervous system (G00-G99) | extrapyramidal disorders (G20)  Alzheimer's disease (G31)  epilepsy (G40 - G41)  migraine (G43)  headache (G44, R51)  TIA (G45 w/out G45.4)  TGA (G45.4)  polyneuropathy (G60 - G63) | 13  6  22  7  9  25  4  29 |
| Chapter VII  Diseases of the eye and adnexa (H00-H59) |  |  |
| Chapter VIII  Diseases of the ear and mastoid process (H60-H95) | Impaired vision (H53)  Disease of the external ear (H60, H65, T16)  Dysfunction of the peripheral vestibular system (H81)  Other reasons for hearing loss (H91)  Otalgia and hearing loss (H92) | 10  17  10  7  4 |
| Chapter IX  Diseases of the circulatory system (I00-I99) | Hypertension (I10)  coronary syndrome (I20 - I21)  heart failure (I25, I42, I50)  thromboembolism / pulmonary embolism (I26-I27, I82)  arrhythmia (R00, I44 - I49)  cerebral haemorrhage (I61)  ischemic disease (I63, I64)  cerebrovascular disease (I67)  sequelae of cerebrovascular disease (I69)  orthostasis (I95, I98) | 10  12  50  21  45  10  10  15  7  12 |
| Chapter X  Diseases of the respiratory system (J00-J99) | acute infection of the upper airway  (J00-J11)  pneumonia (J13 - J18)  bronchitis / COPD (J20 - J44)  asthma (J45) | 37  28  116  5 |
| Chapter XI  Diseases of the digestive system (K00-K93) | ulcer disease (K20 - K29)  disease of the intestines (K50 - K59)  peritonitis (K63 - K65)  liver disease (K70 - K74)  biliary (K80 - K83)  other diseases of the digestive system (K92) | 73  98  24  48  44  6 |
| Chapter XII Diseases of the skin and subcutaneous tissue (L00-L99) | Disease of the skin (L00 - L08) | 40 |
| Chapter XIII  Diseases of the musculoskeletal system and connective tissue (M00-M99) | pyogenic arthritis (M00)  spinal problem (M40 - M48, M53)  back pain (M54)  rhabdomyolysis (M62.8) | 56  531  50  11 |
| Chapter XIV  Diseases of the genitourinary system (N00-N99) | renal failure (N17 - N19)  urinary tract infection (N30, N39) | 37  24 |
| Chapter XV  Pregnancy, childbirth and the puerperium (O00-O99) |  |  |
| Chapter XVI  Certain conditions originating in the perinatal period (P00-P96) |  |  |
| Chapter XVII  Congenital malformations, deformations and chromosomal abnormalities (Q00-Q99) |  |  |
| Chapter XVIII  Symptoms, signs and abnormal clinical and laboratory findings, not elsewhere classified (R00-R99) | Bleeding from the respiratory tract (R04)  breathing disorders (R06)  throat and chest pain (R07)  abdominal and pelvic pain (R10)  nausea and vomiting (R11)  Sensitivity disorder of the skin (R20)  urinary retention (R33)  nonspecific dizziness (R42)  Speech and language disorders (R47)  fever of unknown origin (R50)  pain, not further classified (R52)  syncope and collapse (R55) cramps, not further classified (R56) bleeding, not further classified (R58) | 6  12  6  6  1  6  1  1  4  6  5  1  3  1 |
| Chapter XIX  Injury, poisoning and certain other consequences of external causes (S00-T98) | trauma related (S00-S02, S06-14, T79)  foreign object in respiratory tract (T17)  drug related problems, intoxications and poisoning (F10 - F12, T36 - T47, T50-T51, T65, T78)  surgical complications (T81-T82) | 1452  8  201  22 |
| Chapter XX  External causes of morbidity and mortality (V01-Y98) |  |  |
| Chapter XXI  Factors influencing health status and contact with health services (Z00-Z99) | diagnosis for patient without complaints or known diagnosis (Z00) | 9 |
| Chapter XXII  Codes for special purposes (U00-U99) |  |  |
| *ICD International Classification of Diseases | | |
